# Histone deacetylase (HDAC) inhibitory and antiproliferative activities of phenolic-rich extracts derived from the rhizome of *Hydnophytum formicarum* Jack.: sinapinic acid acts as HDAC inhibitor

**DOI:** 10.1186/1472-6882-13-232

**Published:** 2013-09-22

**Authors:** Thanaset Senawong, Suwatchai Misuna, Somprasong Khaopha, Suporn Nuchadomrong, Prasan Sawatsitang, Chanokbhorn Phaosiri, Arpa Surapaitoon, Banchob Sripa

**Affiliations:** 1 Department of Biochemistry, Faculty of Science, Khon Kaen University, Khon Kaen 40002, Thailand; 2 Department of Chemistry, Faculty of Science, Khon Kaen University, Khon Kaen 40002, Thailand; 3 Department of Pathology, Faculty of Medicine, Khon Kaen University, Khon Kaen 40002, Thailand

**Keywords:** *Hydnophytum formicarum* Jack, HDAC inhibitors, Anticancer activity, Sinapinic acid

## Abstract

**Background:**

The rhizome of *Hydnophytum formicarum* Jack., a medicinal plant known in Thai as Hua-Roi-Roo, has been used in Thai traditional herbal medicine for treatment of cancer. We assessed the ability of its ethanolic and phenolic-rich extracts and its major phenolic compound, sinapinic acid, possessing histone deacetylase (HDAC) inhibitory activity to inhibit proliferation of 5 human cancer cell lines.

**Methods:**

HeLa cells were used to study HDAC inhibitory activity of the extracts, sinapinic acid, and a well-known HDAC inhibitor sodium butyrate. Five human cancer cell lines and one non-cancer cell line were used to study antiproliferative activities of the plant extracts, sinapinic acid and sodium butyrate, comparatively.

**Results:**

Results indicated that ethanolic and phenolic-rich extracts of *H. formicarum* Jack. rhizome possessed both antiproliferative activity and HDAC inhibitory activity in HeLa cells. Sinapinic acid, despite its lower HDAC inhibitory activity than the well-known HDAC inhibitor sodium butyrate, inhibited the growth of HeLa and HT29 cells more effectively than sodium butyrate. However, sinapinic acid inhibited the growth of HCT116 and Jurkat cells less effectively than sodium butyrate. The non-cancer cell line (Vero cells) and breast cancer cell line (MCF-7 cells) appeared to be resistant to both sinapinic acid and sodium butyrate. The growth inhibitory effects of the ethanolic and phenolic-rich extracts and sinapinic acid in HeLa cells were mediated by induction of apoptosis.

**Conclusions:**

The results of this study support the efficacy of *H. formicarum* Jack. rhizome ethanolic and phenolic-rich extracts for the treatment of cervical cancer, colon cancer, and T- cell leukemia in an alternative medicine. Further studies of other active ingredients from this plant are needed.

## Background

In Thailand, a number of plants have been used in Thai traditional herbal medicine for treatment of various malignancies [1-5]. The rhizome of *Hydnophytum formicarum* Jack., a medicinal plant known in Thai as Hua-Roi-Roo, has been used against inflammation and cancer [1-3]. The antiproliferative activities against human cancer cell lines were described [4-7], however, the bioactive ingredients underlying such activity remain to be explored. The screening for histone deacetylase (HDAC) inhibitors from Thai medicinal plants revealed that ethanolic crude extract from the rhizome of *H. formicarum* Jack. possessed HDAC inhibitory activity in vitro [8].

HDAC inhibitors belong to an exciting new class of chemotherapeutic drug currently in several clinical trials with promising results as anticancer agents [9-12]. In general, HDAC inhibitors that act on zinc-dependent HDAC isozymes have three structural characteristics: a zinc-binding moiety, an opposite capping group, and a straight chain alkyl, vinyl or aryl linker connecting the zinc-binding moiety and the capping group [9]. Based on their chemical structures, HDAC inhibitors can be classified into four subtypes: (1) short chain fatty acid; (2) hydroxamic acids; (3) benzamides; and (4) cyclic peptides [13]. Although their mechanisms of action are largely unknown, major consequences usually observed upon treatment with HDAC inhibitors include growth arrest, apoptosis, and inhibition of angiogenesis [9]. Because of their low toxicity, HDAC inhibitors constitute a promising treatment for cancer therapy, especially in combination with other chemotherapeutic agents [14,15]. HDAC inhibitor treatments resulted in cancer cell apoptosis due to a shift in the balance of pro- and anti-apoptotic genes toward apoptosis [15].

In recent years, the development and search for novel HDAC inhibitors have become a popular research focus on discovering safe and effective anticancer agents [16,17]. One promising new source of HDAC inhibitors has been discovered in plant secondary metabolites, specifically phenolic compounds. The phenolic compounds of some plants have been shown to possess HDAC inhibitory activity [16,18,19], however, the HDAC inhibitory activity of phenolic compounds from *H. formicarum* Jack., which may underpin its anticancer activity, has not yet been investigated. In this study, the biological evaluation of HDAC inhibition, antiproliferation and apoptosis induction of cervical cancer cell line (HeLa cells) by ethanolic crude extract and phenolic-rich extract of this plant were reported. Moreover, the identification of sinapinic acid, a known phenolic acid, as a novel HDAC inhibitor was also demonstrated. Antiproliferative activity of sinapinic acid compared with a well-known HDAC inhibitor sodium butyrate on five human cancer cell lines was investigated.

## Methods

### Materials

Dried rhizomes of *H. formicarum* Jack. were obtained from a local herbal shop in Khon Kaen Province, Thailand. The rhizomes were collected during March-May 2008, from Narathiwat Province, Thailand. Taxonomic identification was approved by the Forest Herbarium, Department of National Parks, Wildlife and Plant Conservation, Ministry of Natural Resources and Environment, Bangkok, Thailand. A voucher specimen (voucher number TS09001) is deposited at the KKU Herbarium, Department of Biology, Faculty of Science, Khon Kaen University, Khon Kaen, Thailand. Chemicals and most of the pure standards of phenolic acids were purchased from Sigma-Aldrich Corporation (St. Louis, MO, USA). The pure standards of *m*-hydroxybenzaldehyde and *p*-hydroxybenzoic acid were purchased from Fluka (Buchs, Switzerland) and Acros Organics (Geel, Belgium), respectively.

### Crude ethanolic extraction

Five grams of air-dried ground rhizome were macerated and periodically stirred in 50 ml of absolute ethanol for 48 hours. The suspension was filtered through Whatman No. 4 filter paper and centrifuged at 5,000 rpm for 15 minutes. The supernatant was air-dried to yield an ethanolic crude extract. The residue was reconstituted in dimethyl sulfoxide (DMSO) or ethanol before testing and the solvent was used as a negative control.

### Fractionated solvent extraction

Five grams of air-dried ground rhizome were macerated and periodically stirred in 50 ml of hexane for 48 hours. The suspension was filtered through the filter paper and centrifuged at 5,000 rpm for 15 minutes. The supernatant was air-dried to obtain the hexane soluble fraction. The precipitate remaining from hexane extraction was dispersed, macerated and periodically stirred in 50 mL of ethyl acetate for 48 hours. The ethyl acetate suspension was filtered through the filter paper, centrifuged at 5,000 rpm for 15 minutes, and air-dried to obtain the ethyl acetate soluble fraction. The precipitate remaining from ethyl acetate extraction was dispersed, macerated and periodically stirred in 50 ml of methanol for 48 hours. The methanol suspension was filtered through the filter paper, centrifuged at 5,000 rpm for 15 minutes, and air-dried to obtain the methanol soluble fraction. Each solvent fraction was reconstituted in an appropriate vehicle, DMSO or ethanol, before testing.

### Phenolic extraction

Phenolic extraction was performed by using acidic hydrolysis method [20] with some modifications. Briefly, two hundred milliliters of 70% methanol were added to a beaker containing 10 grams of ground rhizome. The mixture was stirred for 2 hours at room temperature and then filtered through the filter paper. The filtrate was evaporated to 60 ml by a rotary evaporator. The remaining filtrate was added with 50 ml of 2 M NaOH and stirred continuously for 12 hours at room temperature. The mixture was centrifuged at 1,700 g for 20 minutes and then filtered through the filter paper. The supernatant was repeatedly extracted three times with 80 ml of diethyl ether, in which the aqueous phase was collected and the diethyl ether phase was discarded. The aqueous phase was adjusted to pH 1.5 by 10 M HCl and filtered through the filter paper. The filtrate was further extracted by 80 ml of diethyl ether for three times, in which the portion of the diethyl ether was collected. The pooled diethyl ether phase was dehydrated with sodium sulphate (Na_2_SO_4_) anhydrous and then filtered through the filter paper. The filtrate was evaporated to 5 ml using a rotary evaporator and finally evaporated to dryness under a gentle stream of nitrogen.

### Determination of total phenolic content

Total phenolic content in ethanolic crude extract was determined by the Folin-Ciocalteu method as described previously [21]. Gallic acid (Sigma) was used as the standard and the result was calculated as μg Gallic Acid Equivalent (GAE) per mg dry weight of the extract.

### HPLC analysis of phenolic-rich extract

The identification of individual phenolic acids in phenolic-rich extract prepared by phenolic extraction as described above was carried out using a Waters HPLC system, based on matching spectrum and retention times of phenolic acid standards. The phenolic acid standards used were gallic acid, protocatechuic acid, *p*-hydroxybenzoic acid, vanillic acid, caffeic acid, syringic acid, *m*-hydroxybenzaldehyde, *p*-coumaric acid, ferulic acid, and sinapinic acid. The HPLC system consisted of a Waters 600E Multisolvent Delivery system, Waters In-Line degasser AF, a Rheodyne injector with sample loop of 20 μl (180 μg of the extract), and a Waters 2669 photodiode array (PDA) detector. Empower software was used for data acquisition. A Waters system column C18 (3.9 mm i.d. × 150 mm, 5 μm particle diameter) coupled to a guard column was used. The temperature of the column was 25°C and the flow rate of mobile phase was 1.0 ml/minute. The compounds were eluted with a gradient elution of mobile phase A (100% acetronitrile) and B (1% acetic acid in deionized water) where A increased from 3% to 8% in 5 minutes, to 10% by 25 minutes and was maintained at 10% for 20 minutes, then returned to initial condition (3%) in 10 minutes and remained for 5 minutes before next injection. Elutes were detected by the PDA detector at wavelength 280 nm.

### In vitro HDAC inhibition activity assay

HDAC inhibitory activity of the *H. formicarum* Jack. rhizome extracts, sinapinic acid (Sigma) and sodium butyrate (Sigma) was determined by using the Fluor-de-Lys HDAC activity assay kit (Biomol, Enzo Life Sciences International, Inc., Plymount Meeting, PA, U.S.A.). The assay was performed according to the manufacturer’s instructions. Fluorescence was measured using a spectra Max Gemini XPS microplate spectrofluorometer (Molecular Devices, Sunnyvale, CA, U.S.A.) with excitation at 360 nm and emission at 460 nm. Inhibition of HDAC activity was monitored by a decrease in fluorescence signal.

### Cell culture

HeLa and HT29 cells were obtained from the National Cancer Institute, Bangkok, Thailand. Jurkat cells were kindly provided by Dr. M. Leid (Oregon State University, Oregon, U.S.A.). HCT116 and MCF-7 cells were kindly provided by Dr. O. Tetsu (University of California, San Francisco, U.S.A.). Vero cells were kindly provided by Dr. S. Barusrux (Khon Kaen University, Khon Kaen, Thailand). Cells were maintained in RPMI-1640 medium supplemented with 10% fetal bovine serum, penicillin (100 U/ml), and streptomycin (100 μg/ml) (Gibco-BRL). The cells were incubated at 37°C in a humidified atmosphere with 5% CO_2_.

### Antiproliferative activity assay

Cells were seeded in a 96-well plate at cell density of 10^4^ cells/well and incubated for 24 hours. Sample groups were treated with different concentrations of *H. formicarum* Jack. rhizome extracts (0.5-3.0 mg/mL), sinapinic acid (0.5-3.0 mM), or sodium butyrate (0.5-3.0 mM) for 24, 48 and 72 hours. Vehicle control groups were added with DMSO (final concentration of 0.05%) or double distilled water. Cell proliferation assays were performed using a WST-8 Cell Proliferation Assay Kit (BioVision, Mountain View, CA, U.S.A.) according to the manufacturer’s instructions. Absorbance (A) was measured at 415 nm using a microtiter plate reader (Bio-Rad Laboratories, Hercules, CA, U.S.A.). The absorbance at 655 nm was used as a reference wavelength. Cell proliferation or cell growth was determined as a percentage of the vehicle control by an equation of;

$$\begin{array}{ll} \%\;\mathrm{Cell}\;\mathrm{Proliferation}= & \left[\left(\mathrm{control}\;A‒\mathrm{sample}\;A\right)/\mathrm{control}\;A\right]\\ & \times 100. \end{array}$$

### Extraction of histone proteins

Cells grown in a 4.5-cm dish were treated with either solvent control or the sample for 6 hours, and the histone proteins were then isolated according to the Abcam’s protocol (Abcam Inc., Cambridge, MA, U.S.A.) with some modifications. In brief, cells were harvested by trypsinization, washed with PBS, and then resuspended in Triton Extraction Buffer (TEB; PBS containing 0.5% (v/v) Triton X-100, 2 mM phenylmethylsulfonyl fluoride, 0.02% (w/v) NaN_3_) at a cell density of 10^5^ cells/ml. The cells were incubated on ice and agitated periodically for 10 minutes. The suspension was centrifuged at 7,500 rpm for 10 minutes at 4°C to spin down the nuclei and the supernatant was discarded. The nuclei pellet was resuspended in 0.2 M HCl at a density of 10^6^ nuclei/ml and incubated overnight at 4°C. The suspension was centrifuged at 7,500 rpm for 10 minutes at 4°C and the supernatant containing histone proteins was collected. Protein concentration was measured by using a Bio-Rad protein assay kit (Bio-Rad Laboratories, Hercules, CA, U.S.A.) based on the Bradford method.

### Acid-Urea Triton X-100 polyacrylamide gel electrophoresis (AUT-PAGE)

Inhibition on acetylation of cellular histones was analyzed by gel electrophoresis using acid urea/Triton-X-100 (AUT) gels [22]. The upper gel consisted of 5% acrylamide/bis-acrylamide containing 0.9 M acetic acid, 8 M urea. The resolving gel was 15% acrylamide/bis-acrylamide containing 0.9 M acetic acid, 8 M urea, and 0.37% Triton X-100. The running buffer was 0.9 M acetic acid. In this buffer system, positively charged proteins migrate toward the cathode. Electrophoresis was performed in a Mini PAGE System (Select BioProducts, Edison, NJ, U.S.A.). Gels were pre-run at 150 volts for 4 hours at the ambient temperature. Wells were then loaded with the second pre-run solution (1 M cysteamine (Sigma-Aldrich), 8 M urea, 0.9 M acetic acid) to scavenge the residual free radicals and the gel was pre-run at 150 volts for a further 40 minutes. Histone samples (5 μg histone protein) solubilized in loading buffer (8 M urea, 10% glycerol, 0.9 M acetic acid, 5% β-mercaptoethanol and 0.25% methylene blue) were boiled for 5 minutes before being loaded and gels were run at 90 volts for 6 hours. Gels were silver-stained by using PageSilver™ Silver Staining Kit (Fermentas, Burlington, Ontario, Canada), dried, and photographed.

### Apoptosis analysis

Apoptosis analysis was performed by using a Vybrant Apoptosis Assay Kit #2 (Molecular Probes, Invitrogen Corporation, Carlsbad, CA, U.S.A.) according to the manufacturer’s instructions. Briefly, cells were seeded at 1.2 × 10^6^ cells/4 ml in a 4.5-cm dish, incubated for 24 hours, and treated with different concentrations of the extracts or sinapinic acid for 6 hours. Cells were harvested by trypsinization, washed with cold PBS, and resuspended in the Annexin-binding buffer. Cell density was determined and diluted in the annexin-binding buffer to 10^5^ cells per assay. Cells were incubated with Alexa Fluor 488 Annexin V and Propidium iodide (PI) at room temperature for 15 minutes. Following the incubation, cells were analyzed by flow cytometry using a Beckman Coulter Cytomics FC500 MPL flow cytometry (Beckman Coulter, Miami, FL, U.S.A.). The flow cytometry results were confirmed by viewing the cells under a fluorescence microscope.

### Statistical analysis

Data are expressed as means ± standard deviation (SD) from three independent experiments. Tests for significant differences between vehicle controls and sample treated cells were carried out using one-way ANOVA with Duncan’s post hoc test. The criterion for statistical significance was set at *p* <0.05.

## Results

### In vitro HDAC inhibitory activity of the extracts from *H. formicarum* Jack. rhizome

**Figure 1:**
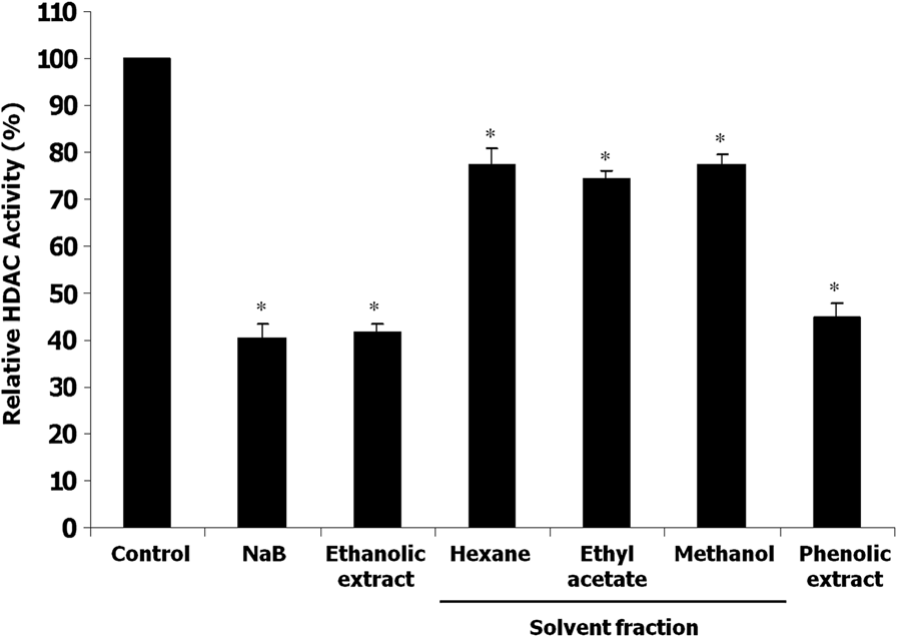
**In vitro HDAC inhibitory activity of ethanolic crude extract, solvent extraction fractions, and phenolic-rich extract.** The concentration of each sample in a reaction mixture was 200 μg/50 μl. Results were expressed as relative HDAC activity with respect to the vehicle control (DMSO). Sodium butyrate (NaB) was used as a positive control. Each value represents the mean ± SD of three experiments, performed in duplicate. Asterisks (*) denote significant differences *vs* control (*p* < 0.05).

The effect of various polarity extracts including fractionated solvent extracts from hexane soluble fraction, ethyl acetate soluble fraction, methanol soluble fraction and also ethanolic crude extract on in vitro HDAC activity was examined by using HeLa nuclear extract as a source of the HDAC enzymes. As shown in Figure 1, all of the above mentioned extracts significantly inhibited HDAC activity (*p* < 0.05). Among various polarity extracts tested, ethanolic crude extract exhibited the most potent HDAC inhibition of 55.2 ± 3.2% as compared to the control. Therefore, this extract was used to investigate the further effects of this plant on cancer cells.

Several lines of evidence indicate that some plant phenolic compounds possess HDAC inhibitory activity [16,18,19]. Therefore, we intended to investigate the effect of phenolic extract from *H. formicarum* Jack. rhizome on HDAC activity in vitro. As expected, phenolic extract of this plant significantly inhibited HDAC activity (*p* < 0.05), and its effect was comparable to that of the ethanolic crude extract (Figure 1). The presence of phenolic compounds in the ethanolic crude extract was verified by the Folin-Ciocalteu reaction and total phenolic content was 316.28 ± 12.18 μg Gallic Acid Equivalent (GAE)/mg dry weight. Because phenolic-rich extract was found to possess HDAC inhibitory activity, therefore, this extract was also used to investigate the further effects on cancer cells.

### Sinapinic acid is a major phenolic acid of *H. formicarum* Jack. rhizome possessing HDAC inhibitory activity

**Figure 2:**
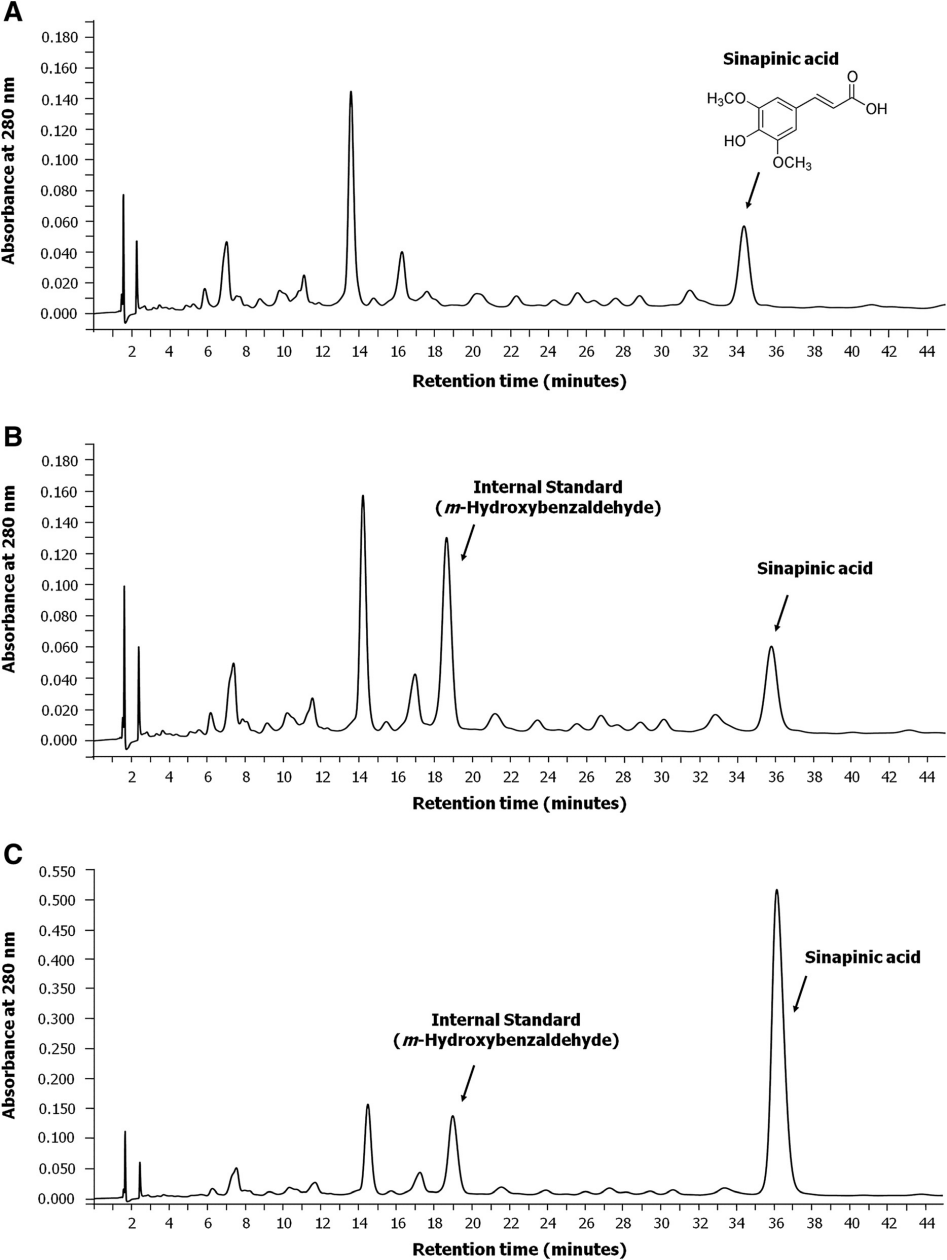
**HPLC profiles of phenolic-rich extract from *H. formicarum *Jack. rhizome.** HPLC profiles of **(A)** phenolic-rich extract alone (180 μg), **(B)** phenolic-rich extract added with the internal standard, *m*-hydroxybenzaldehyde (1 μg), and **(C)** phenolic-rich extract added with the internal standard (1 μg) and the standard sinapinic acid (2 μg), are displayed comparatively. The data shown are representative of two independent experiments performed in duplicate. A phenolic compound, sinapinic acid, is indicated and its chemical structure is depicted.

**Figure 3:**
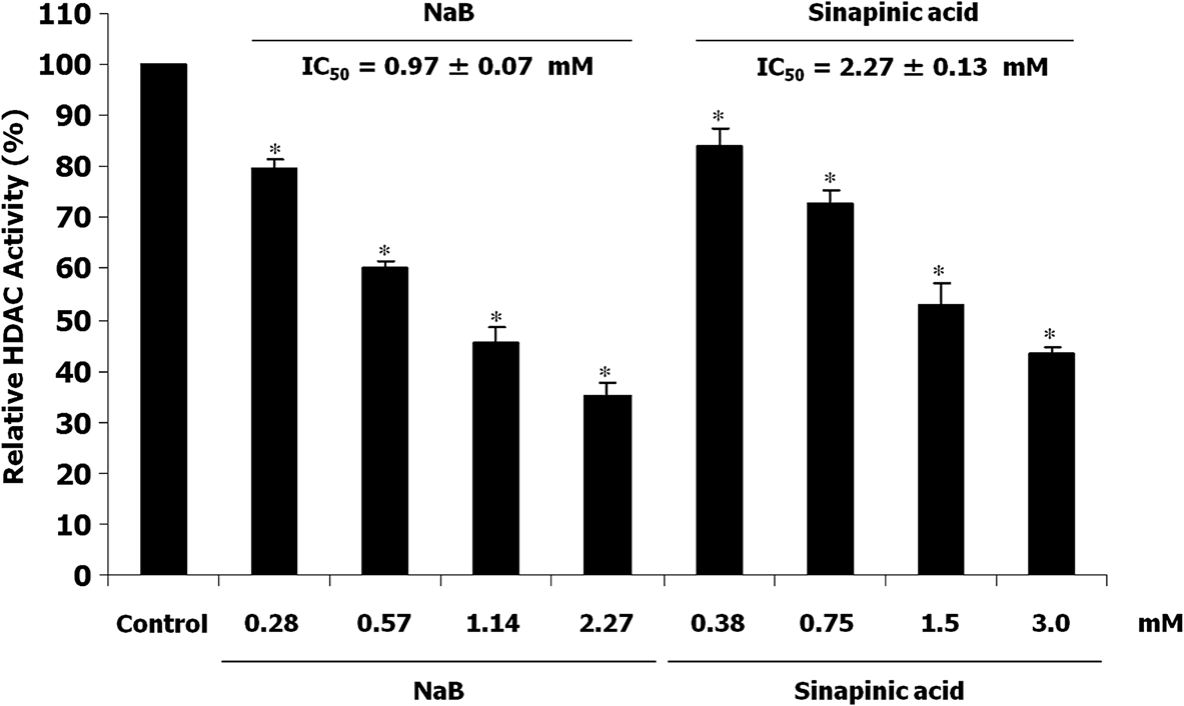
**In vitro HDAC inhibitory activity of sinapinic acid.** Results were expressed as relative HDAC activity with respect to the vehicle control (DMSO). Sodium butyrate (NaB) at indicated concentrations was studied comparatively. Each value represents the mean ± SD of three experiments, performed in duplicate. Asterisks (*) denote significant differences *vs* control (*p* < 0.05). Half maximal inhibitory concentration (IC_50_) values represent concentrations of the indicated compounds that inhibit 50% of HDAC activity in vitro.

Some phenolic compounds were previously discovered in the crude ethyl acetate extract of this plant [7], however, their HDAC inhibitory activity has not yet been explored. Preliminary separation and identification of individual phenolic compounds in phenolic extract was conducted by the reversed phase HPLC. Identification of sample peaks by matching against retention time and spectra of known phenolic standards under the same chromatographic conditions revealed that sinapinic acid was one of the two major components of phenolic-rich extract of *H*. *formicarum* Jack. rhizome (Figure 2A, 2B). The confirmation of peak was obtained by the addition of sinapinic acid standard into the sample for HPLC analysis (Figure 2C). The yield of phenolic-rich extract from 10 g of *H. formicarum* Jack. rhizome was 67.5 mg. The amount of sinapinic acid was 3.4 μg/mg of phenolic-rich extract. However, other sample peaks remained to be identified. Interestingly, sinapinic acid was found to act as HDAC inhibitor, blocking the enzyme activity in vitro with an IC_50_ value (2.27 ± 0.13 mM) higher than that of the well-known HDAC inhibitor sodium butyrate (0.97 ± 0.07 mM) (Figure 3). These findings suggest that sinapinic acid takes account, at least in part, for the inhibition of HDAC activity by the plant phenolic extract (Figure 1).

### Ethanolic crude extract, phenolic-rich extract and sinapinic acid inhibit HDAC activity in HeLa cells

**Figure 4:**
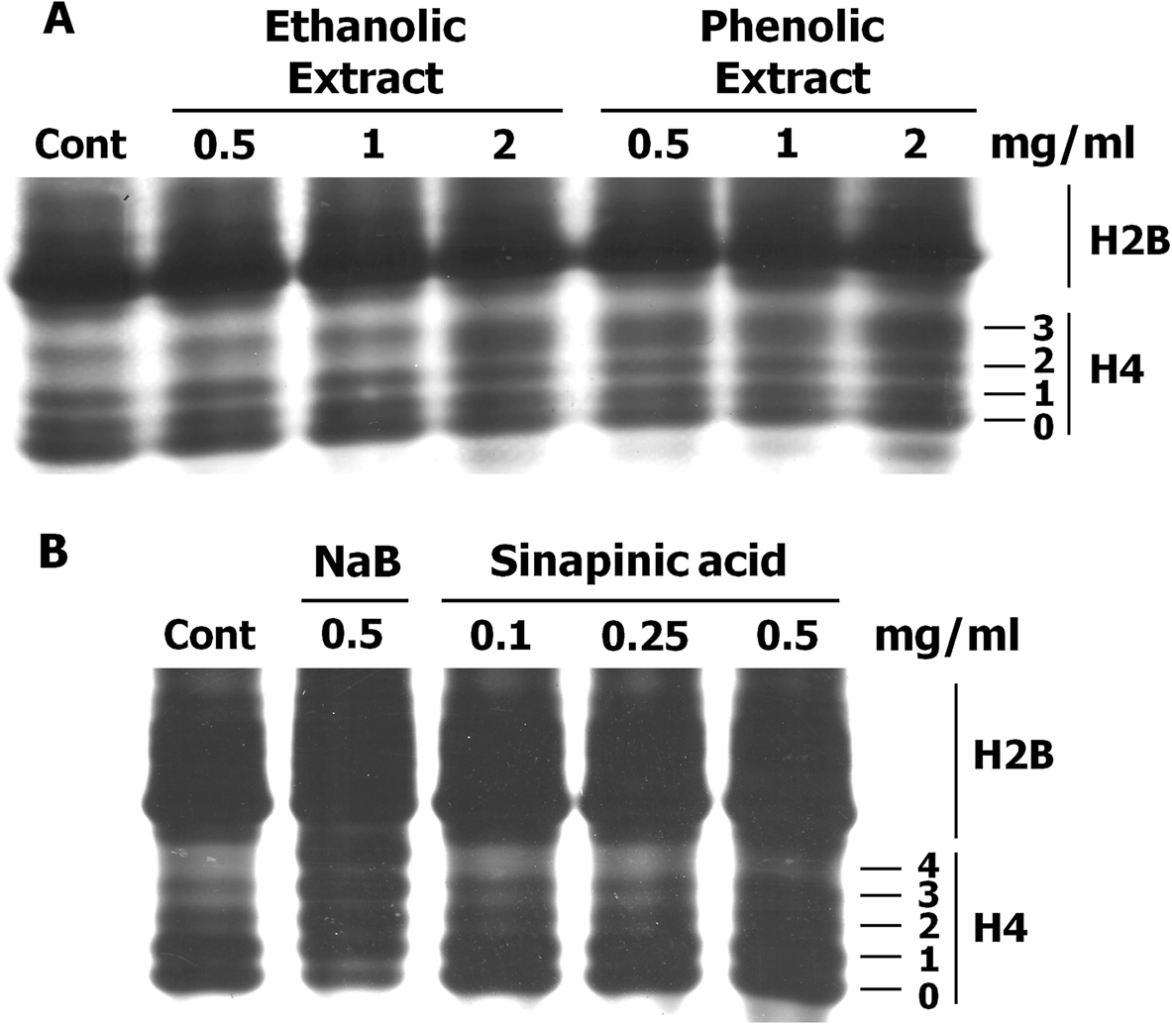
**HDAC inhibitory activity of the plant extracts and sinapinic acid in HeLa cells.** Effect of **(A)** ethanolic crude extract, phenolic-rich extract and **(B)** sinapinic acid on histone acetylation in HeLa cells. After 6 h-treatment, the level of histone acetylation was determined by AUT gel electrophoresis as described in *Materials and Methods*. Control (Cont) represents the level of histone acetylation in vehicle control treatment. The sodium butyrate (NaB)-treatment was used as a positive control. The degree of histone acetylation of histone H4 is indicated as follows: 0, nonacetylated; 1, monoacetylated; 2, diacetylated; 3, triacetylated; and 4, tetraacetylated. The data shown are representative of two independent experiments performed in duplicate.

HDAC inhibition by ethanolic crude extract, phenolic-rich extract and sinapinic acid in HeLa cells was analyzed by AUT gel electrophoresis, whereby each cellular core histone (H2A, H2B, H3, and H4) with different extent of acetylation can be separated. Herein, the profiles of histones H4 and H2B extracted from ethanolic crude extract-, phenolic-rich extract-, or sinapinic acid-treated HeLa cells were demonstrated (Figure 4). The addition of ethanolic crude extract and phenolic extract to cell cultures resulted in the accumulation of hyperacetylated histone H4 molecules, which could be detected clearly on AUT gel (Figure 4A). The histone H4 with three acetylated lysine residues was markedly increased when treated the cells with ethanolic and phenolic-rich extracts (Figure 4A). Similarly, treatment of HeLa cells with sinapinic acid clearly increased di- and tri-acetylated H4 molecules with two and three acetylated lysine residues, respectively (Figure 4B). However, HDAC inhibition of sinapinic acid in the cell was much less effective when compared to that of sodium butyrate. These observations indicated that ethanolic crude extract, phenolic-rich extract and sinapinic acid inhibited HDAC activity not only in vitro but also in the cells.

### Effect of ethanolic crude extract, phenolic-rich extract and sinapinic acid on proliferation of human cancer cell lines

The anticancer activity of the two rhizome extracts and sinapinic acid was further investigated in 5 human cancer cell lines and in a non cancer cell line (Vero cells). As shown in Table 1, ethanolic and phenolic-rich extracts possessing HDAC inhibitory activity inhibited the growth of HeLa cells in a dose- and time-dependent manner with IC_50_ values of 0.54 ± 0.03 and 0.30 ± 0.05 mg/ml, respectively, for exposure time of 72 hours. Phenolic-rich extract showed greater antiproliferative activity than ethanolic crude extract on growth inhibition of HeLa cells. However, both extracts showed no significant activity on non cancer cells and other cancer cell lines tested. Sinapinic acid significantly inhibited the growth of HeLa cells with an IC_50_ value (0.91 ± 0.02 mM) lower than sodium butyrate (1.22 ± 0.03 mM) for exposure time of 72 hours. Sinapinic acid also showed greater antiproliferative activity than sodium butyrate on HT29 cells. The antiproliferative activity of sinapinic acid against HCT116 cells was not significantly different from that of sodium butyrate. In contrast, sinapinic acid showed a less efficient activity than sodium butyrate against Jurkat cells. Further, both sinapinic acid and sodium butyrate showed no significant activity on non cancer and breast cancer (MCF-7) cell lines. This finding suggests that sinapinic acid may underpin, at least in part, both the HDAC inhibitory activity and anticancer activity of the rhizome extracts.

**Table 1:** Antiproliferative activities of the plant extracts and sinapinic acid on human cancer cell lines

**Extracts/Compounds**	**Molecular weight (Dalton)**	**Structure**	**IC**_**50 **_**values**^**a **^**(mean ± SD; *n *= 3; mg/mL for the plant extracts; mM for the compounds)**																	
			**Vero cells**^**b**^			**HeLa cells**			**HT29 cells**			**HCT116 cells**			**MCF-7 cells**			**Jurkat cells**		
			**24 h**	**48 h**	**72 h**	**24 h**	**48 h**	**72 h**	**24 h**	**48 h**	**72 h**	**24 h**	**48 h**	**72 h**	**24 h**	**48 h**	**72 h**	**24 h**	**48 h**	**72 h**
Ethanolic extract	-	-	>3	>3	>3	>3	2.6 ± 0.02	0.5 ± 0.03	>3	>3	>3	>3	>3	>3	>3	>3	>3	>3	>3	>3
Phenolic-rich extract	-	-	>3	>3	>3	2.20 ± 0.03	1.72 ± 0.03	0.3 ± 0.05	>3	>3	>3	>3	>3	>3	>3	>3	>3	>3	>3	>3
Sinapinic acid^c^	224.21		>3	>3	>3	2.35 ± 0.03	2.04 ± 0.06	0.9 ± 0.02	2.97 ± 0.05	2.2 ± 0.03	1.6 ± 0.02	>3	2.2 ± 0.15	2.1 ± 0.06	>3	>3	>3	>3	>3	2.8 ± 0.12
Sodium butyrate^d^	110.09		>3	>3	>3	2.63 ± 0.06	2.22 ± 0.13	1.2 ± 0.03	>3	>3	2.57 ± 0.03	>3	2.2 ± 0.20	2.0 ± 0.05	>3	>3	>3	>3	2.36 ± 0.19	1.5 ± 0.30

^a^IC_50_ values represent concentrations of the indicated extracts/compounds that inhibit 50% of cell proliferation.

^b^A non cancer cell line.

^c^The chemical structure of its prototype, a cinnamic acid, is shown here. .

^d^A well known HDAC inhibitor.

### Induction of apoptosis by ethanolic crude extract, phenolic extract and sinapinic acid in HeLa cells

Histone acetylation leads to modulation of expression of a specific set of genes that result in cell cycle arrest and induction of apoptosis [23,24]. HDAC inhibitors induce apoptosis in a number of tumor cell types and through various mechanisms [25]. To investigate the mechanism of antiproliferative effect of ethanolic crude extract, phenolic extract and sinapinic acid on HeLa cells, we examined their capacity to induce apoptosis. Apparently, ethanolic crude extract, phenolic extract, and sinapinic acid exhibited a significant effect on induction of apoptosis in HeLa cells even only 6 hours of exposure time (Table 2). The treatment of HeLa cells with 1.4 mg/ml of ethanolic and phenolic-rich extracts resulted in the increase of early apoptotic cells up to 42.9% and 78.9%, respectively. The treatment with 9 mM of sodium butyrate and sinapinic acid resulted in the increase of early apoptotic cells up to 7.6% and 8.4%, respectively. In contrast, the control HeLa cells had only 0.95% of apoptotic cells. These results suggest that ethanolic crude extract and phenolic extract, as well as sinapinic acid, suppress the HeLa cell growth through induction of apoptosis.

**Table 2:** Induction of apoptosis by the plant extracts and sinapinic acid in HeLa cells

**Apoptotic cells**	**Extracts/Compounds**									
	**Solvent control (DMSO; 0.05%)**	**Positive control (Camptothecin; 0.1 mg/mL)**	**Ethanolic extract**		**Phenolic-rich extract**		**Sodium butyrate**		**Sinapinic acid**	
			**0.7 mg/mL**	**1.4 mg/mL**	**0.7 mg/mL**	**1.4 mg/mL**	**4.5 mM**	**9.0 mM**	**4.5 mM**	**9.0 mM**
Number of Early Apoptotic cells (%)	0.95 ± 0.35	91.65 ± 3.75	6.40 ± 3.11	42.90 ± 4.81	20.70 ± 6.65	78.90 ± 5.09	3.75 ± 1.06	7.60 ± 1.27	4.40 ± 1.27	8.40 ± 1.55
Number of Late Apoptotic cells (%)	0.20 ± 0.14	3.55 ± 1.06	2.05 ± 0.92	4.15 ± 0.07	3.50 ± 1.84	4.15 ± 2.05	1.00 ± 0.28	2.05 ± 1.06	1.75 ± 0.78	3.25 ± 1.06

Cells labeled with Alexa Fluor 488 Annexin V and Propidium iodide (PI) after 6 h-treatment were analyzed by flow cytometry. Each value represents the mean ± SD of three experiments performed in duplicate.

## Discussion

An expensive cost of cancer chemotherapy is a big problem for patients in developing countries. Therefore, an alternative medicine for cancer treatment is still an inevitable option in low-income countries. While many poor patients in these countries still struggle to save their life with the use of traditional medicinal plants where most of the plant’s active ingredients remains to be investigated. To our knowledge, this is the first time that sinapinic acid, a derivative of cinnamic acids, is identified as an HDAC inhibitor. However, HDAC inhibition of sinapinic acid in the cell context was much less effective than that of sodium butyrate. This might be due to the greater difficulty of water soluble property of sinapinic acid or there might be some structural changes during transportation in a cell. Indeed, sinapinic acid has a partition coefficient (log *P*) value (log *P* = 1.52; ChemAxon) greater than that of sodium butyrate (log *P* = 0.92; ChemAxon), indicating its difficulty of water solubility than sodium butyrate. The two methoxyl groups at C3 and C5 positions of sinapinic acid (Table 1) have little influence on its hydrophobicity while the hydroxyl group at C4 position contributes to a lesser extent of its hydrophobicity comparing to the prototype cinnamic acid (log *P* = 2.14; ChemAxon).

In consistence with our results, it has been reported that two other members of cinnamic acids, *p*-coumaric acid and caffeic acid, possess in vitro HDAC inhibitory activity [26], however, their HDAC inhibitory activity in mammalian cells has not yet been reported. Further investigation on the role of various cinnamic acids in HDAC inhibition and anticancer action would be of interest to constitute a novel group of HDAC inhibitors. Similar to HDAC inhibitors in the short-chain fatty acid group [26,27], HDAC inhibitors of the proposed cinnamic acid group seem to be effective at millimolar concentrations in vitro. Since we observed HDAC inhibitory activity in several polarity extracts tested (Figure 1), it is hopeful that HDAC inhibitors other than sinapinic acid remain to be identified from this plant.

A nuclear extract of HeLa cells was a rich source of HDAC enzymes. Currently, eighteen HDACs have been established in humans, and they are grouped into four classes based on their homology to yeast HDACs, their enzymatic activities and their subcellular localization [9,24,27]. As shown in Figure 4A, a markedly increase in tri-acetylated H4 molecules was observed after the cells were treated with ethanolic crude extract and phenolic extract. This particular hyperacetylation pattern is different from that of sodium butyrate- and sinapinic acid-induced acetylated histone H4 (Figure 4B). This discrepancy may be explained by a different sensitivity of specific HDAC(s) to the inhibitor(s) [28] and/or a different mechanism, reversible or irreversible, of HDAC inhibition by the inhibitors [27]. Further studies are needed to elucidate the specificity of the above mentioned extracts and sinapinic acid for individual HDAC family members.

Based on our findings that sinapinic acid possesses antiproliferative activity more effective than a well-known HDAC inhibitor sodium butyrate against HeLa and HT29 cells (Table 1), one may envision a role for sinapinic acid in a HDAC inhibitor-based cancer treatment. Although antiproliferative activities of the plant extracts and sinapinic acid were not appreciably potent for a single-drug treatment, further investigation on the use of sinapinic acid or the plant extracts in combination with other anticancer drugs/medicinal plants may enable the development of more effective therapeutic strategies. The low efficient antiproliferative activity of the plant extracts (Table 1) may be due to the presence of some phenolic antioxidants [7]. Antioxidant activity of sinapinic acid was observed at low concentrations [29], whereas its antiproliferative activity was observed at higher concentrations (Table 1). Despite its low efficient antiproliferative activity, sinapinic acid possesses HDAC inhibitory activity making it more attractive in combination chemotherapy. In this regard, combining HDAC inhibitor vorinostat with aurora kinase inhibitors enhances cancer cell killing [30], and combining HDAC inhibitor sodium butyrate with Doxorubicin potentiates apoptosis of myeloma cells [31].

Theoretically, our findings may validate the use of *H. formicarum* Jack. rhizome extracts in combination with other plant extracts as an alternative medicine for cancer treatment [1-3].

## Conclusions

The results in this report demonstrated that ethanolic crude extract and phenolic-rich extract from *H. formicarum* Jack. rhizome inhibited HDAC activity both in vitro and in the cells. Sinapinic acid was identified as the major component of phenolic extract, which may underpin, at least in part, its HDAC inhibitory activity. The growth inhibitory effect on a cervical cancer cell line (HeLa cells) of ethanolic crude extract, phenolic extract and sinapinic acid is in accordance with their capability to induce cancerous cell apoptosis. Our findings may validate the use of *H. formicarum* Jack. rhizome extracts as an alternative medicine for cancer treatment. Further investigation, with details about chemical structure modification of sinapinic acid, HDAC inhibitory activity, anticancer activity and combination with other anticancer drugs, is of interest.

## Abbreviations

HDAC: Histone deacetylase; H. formicarum Jack.: *Hydnophytum formicarum* Jack.; AUT: Acid Urea/Triton-X-100; DMSO: Dimethyl sulfoxide; NaB: Sodium butyrate; PI: Propidium iodide.

## Competing interests

The authors declare that they have no competing interests.

## Authors’ contributions

TS conceived the study, directed its design and coordination, and manuscript preparation. SM did all plant extractions and HDAC activity assays. SK did the antiproliferation and apoptosis assays. SN directed preparation of plant extracts and manuscript editing. PS directed determination of total phenolic content. CP provided the materials for plant extraction. AS directed the use of flow cytometer and analyzed apoptosis data. BS provided materials for apoptosis assay. All authors read and approved the final manuscript.

## Pre-publication history

The pre-publication history for this paper can be accessed here:

http://www.biomedcentral.com/1472-6882/13/232/prepub
